# Correction: Correlation between musculoskeletal structure of the hand and primate locomotion: Morphometric and mechanical analysis in prehension using the cross- and triple-ratios

**DOI:** 10.1371/journal.pone.0233867

**Published:** 2020-05-21

**Authors:** The PLOS ONE Staff

S2 Fig is a duplicate of S3 Fig. Please view the correct S2 Fig below. The publisher apologizes for the error.

## Supporting information

S2 Fig Relationship between the joint torque and bone length in a simple joint model. The holding torque is proportional to the square of the length of the proximal phalanx. *b*, length of the proximal phalanx; *f*, reaction force (thick arrows) from the central axis of the cylinder to the bone; *r*, radius of the cylinder; *θ*, the angle between *f* and *x*-axis; τ_*s*_, joint torque.
(TIF)
