# Supplementary figures and images for: Correlation between musculoskeletal structure of the hand and primate locomotion: Morphometric and mechanical analysis in prehension using the cross- and triple-ratios
Source: PLoS One. 2020 May 4;15(5):e0232397. doi: 10.1371/journal.pone.0232397 (PMC7197777; doi:10.1371/journal.pone.0232397)

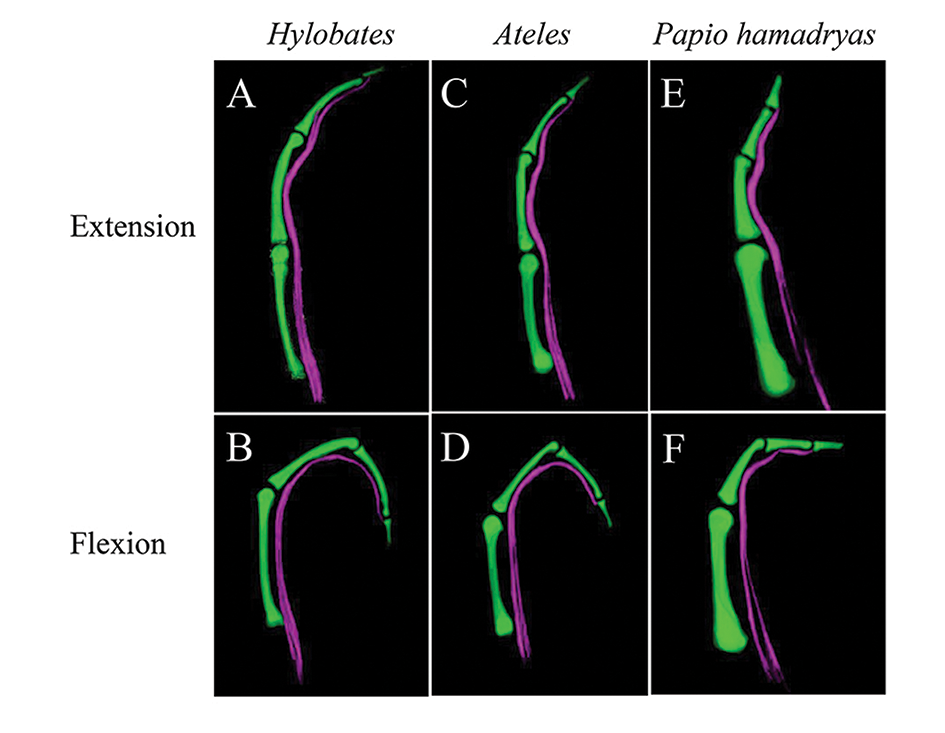

Supplement: S1 Fig — In Hylobates spp. (A, B), Ateles sp. (C, D), and Papio hamadryas (E, F), the phalanges, metacarpal bones, and flexor digitorum tendons were reconstructed from MR images of digit III. Their lateral views at extension (A, C, E) and flexion (B, D, F) are shown. The positional relationship between bones and tendons was changed by the finger posture. (TIF) [file pone.0232397.s001.tif]

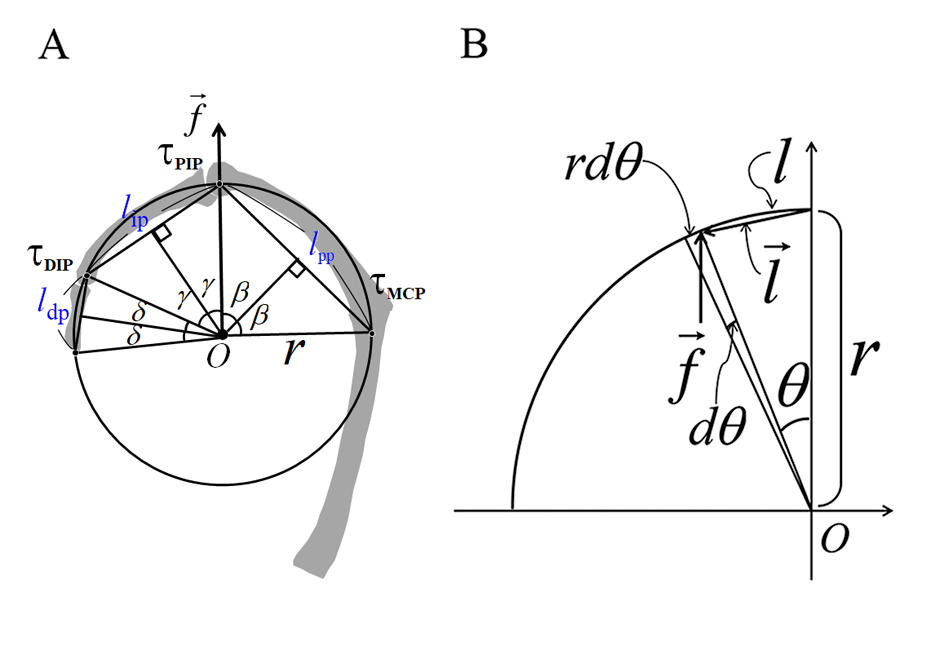

Supplement: S2 Fig — The holding torque is proportional to the square of the length of the proximal phalanx. b, length of the proximal phalanx; f, reaction force (thick arrows) from the central axis of the cylinder to the bone; r, radius of the cylinder; θ, the angle between f and x-axis; τs, joint torque. (TIF) [file pone.0232397.s002.tif]

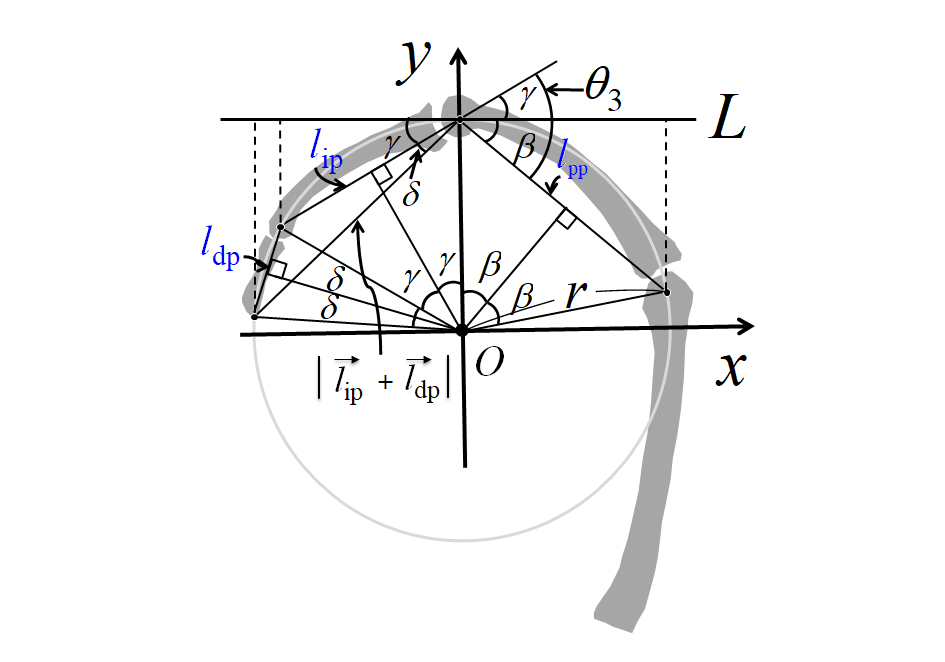

Supplement: S4 Fig — The center and radius of the cylinder are defined as O, and r, respectively. θ3, proximal interphalangeal (PIP) joint angle; lpp, lip, and ldp, the lengths of the proximal, middle, and distal phalanges; β, γ, and δ, inscribe angles of lpp, lip, and ldp; L, the line, which is parallel with the horizontal line (x-axis) and passes through the center of the proximal interphalangeal joint. (TIF) [file pone.0232397.s004.tif]

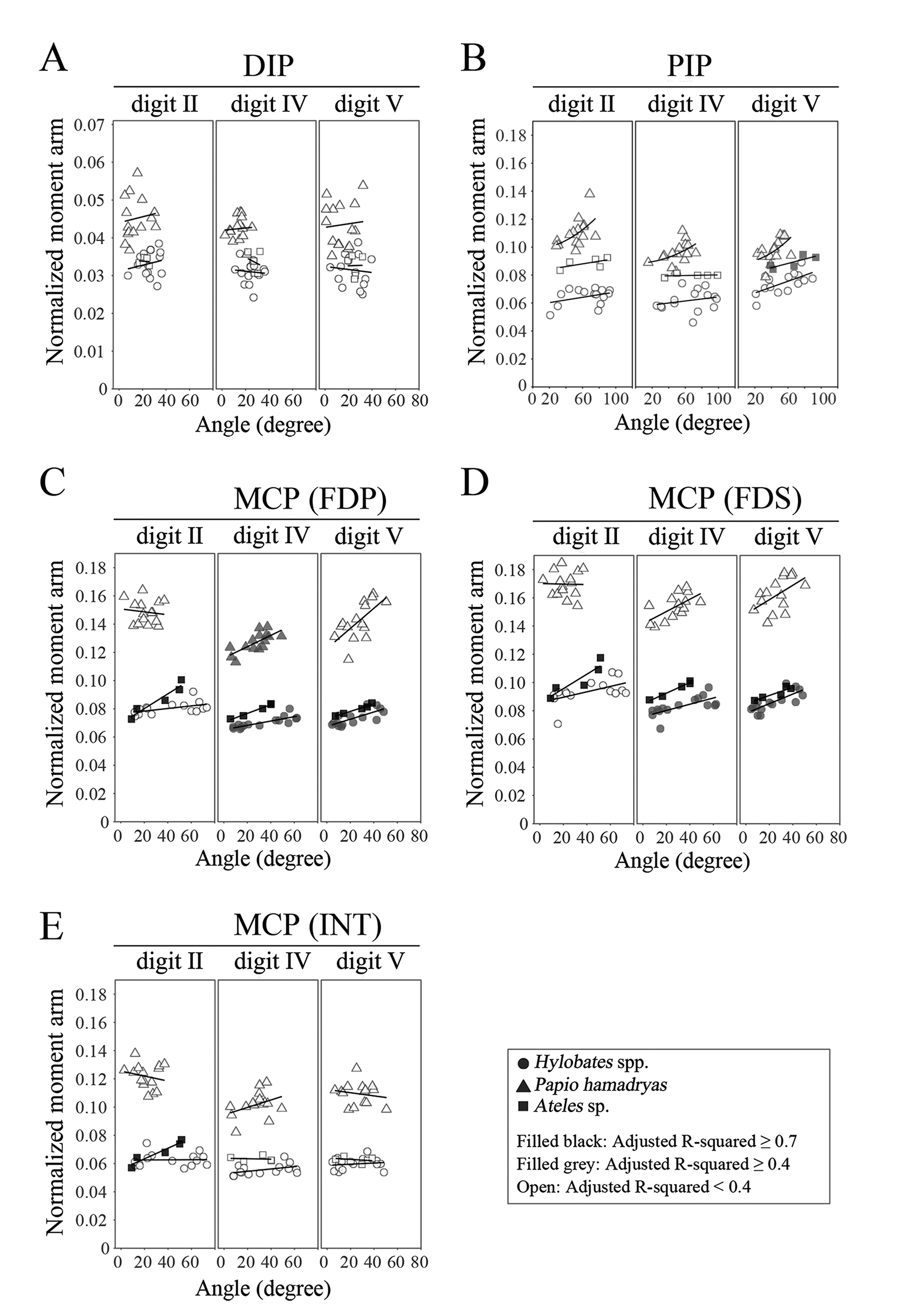

Supplement: S5 Fig — Normalized moment arm, lmL, was calculated on distal interphalangeal (DIP), proximal interphalangeal (PIP), and metacarpophalangeal (MCP) joints. Regression equations are shown in S3 Table. (TIF) [file pone.0232397.s005.tif]

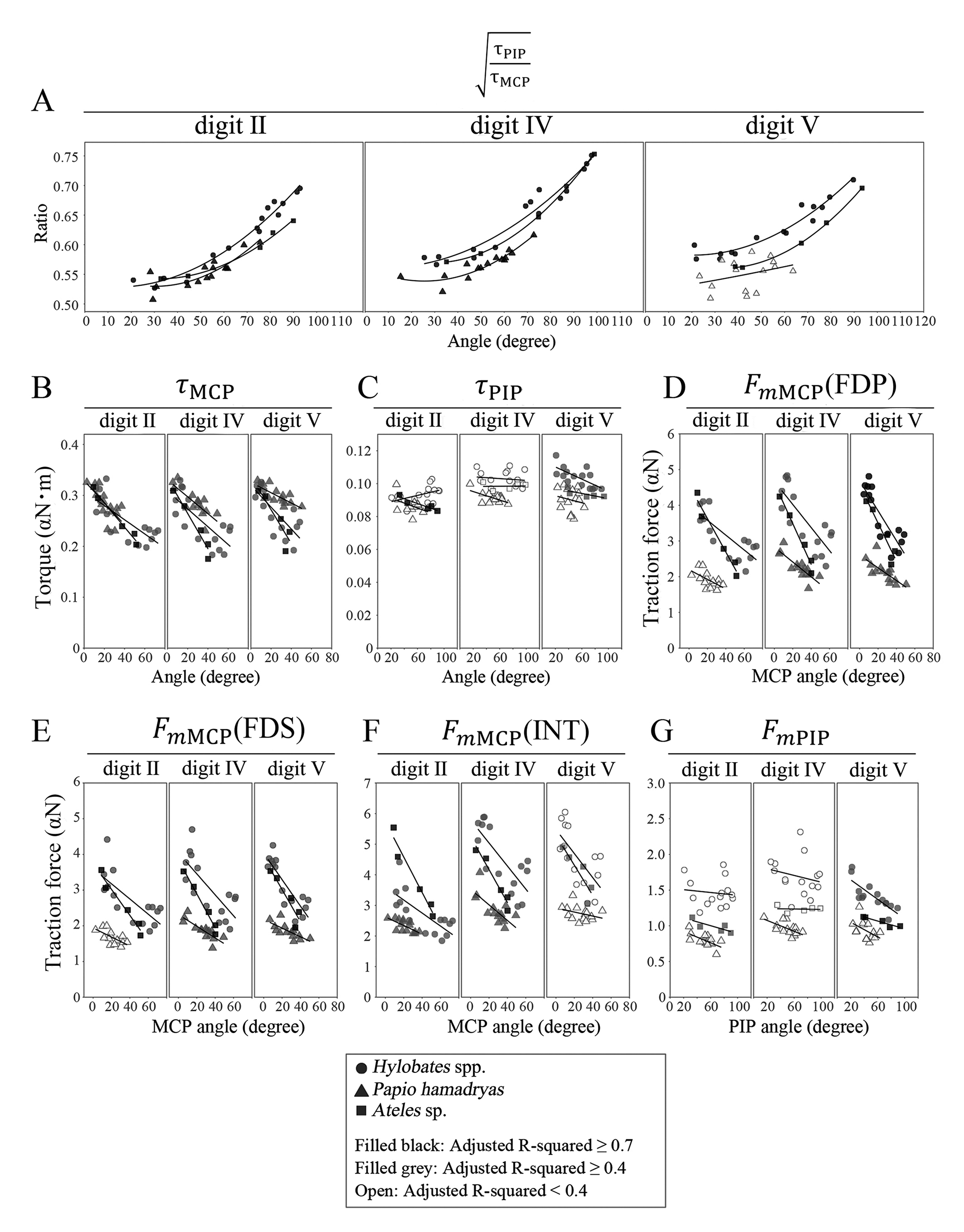

Supplement: S6 Fig — The differences in τPIPτMCP (A), τMCP (B) and τPIP (C), FmMCP of FDP (D), FDS (E) or intrinsic muscles (F), and FmPIP (G) were compared among Hylobates spp., Papio hamadryas, and Ateles sp. using generalized estimating equations (GEE). Circles, triangles, and squares show the data of Hylobates spp., Papio hamadryas, and Ateles sp., respectively. Symbols filled with black, R2 of the regression ≥0.7; symbol filled with gray, R2 of the regression ≥0.4; open symbol, R2 of the regression <0.4. Regression equations are shown in S4 and S6 Tables. (TIF) [file pone.0232397.s006.tif]

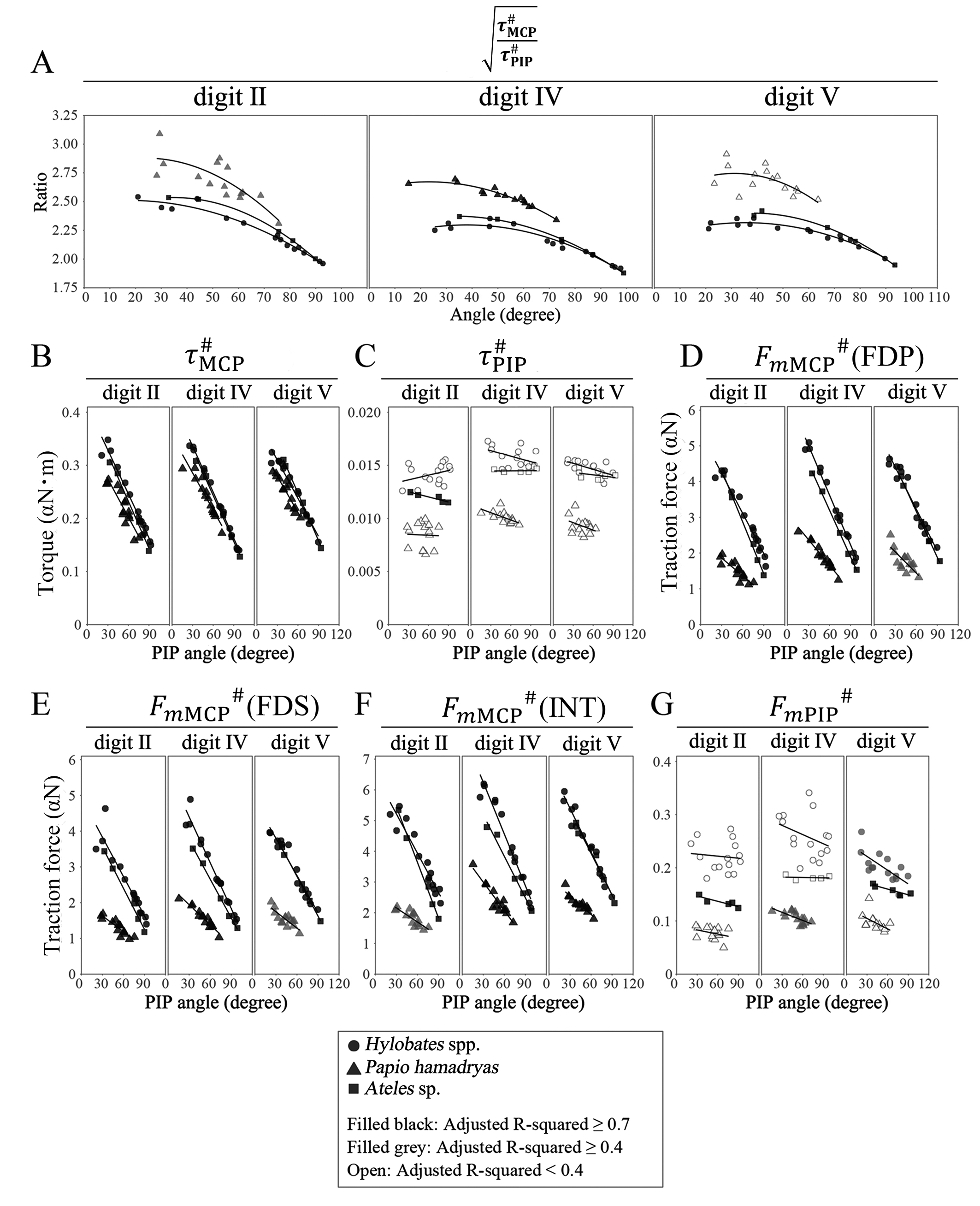

Supplement: S7 Fig — The differences in τMCP#τPIP# (A),τMCP# (B) τPIP#, (C), FmMCP# of FDP (D), FDS (E), or intrinsic muscles (F), and FmPIP# (G) were compared among Hylobates spp., Papio hamadryas, and Ateles sp. using generalized estimating equations (GEE). Circles, triangles, and squares show the data of Hylobates spp., Papio hamadryas, and Ateles sp., respectively. Symbols filled with black, R2 of the regression ≥0.7; symbol filled with gray, R2 of the regression ≥0.4; open symbol, R2 of the regression <0.4. Regression equations are shown in S6 and S7 Tables. (TIF) [file pone.0232397.s007.tif]

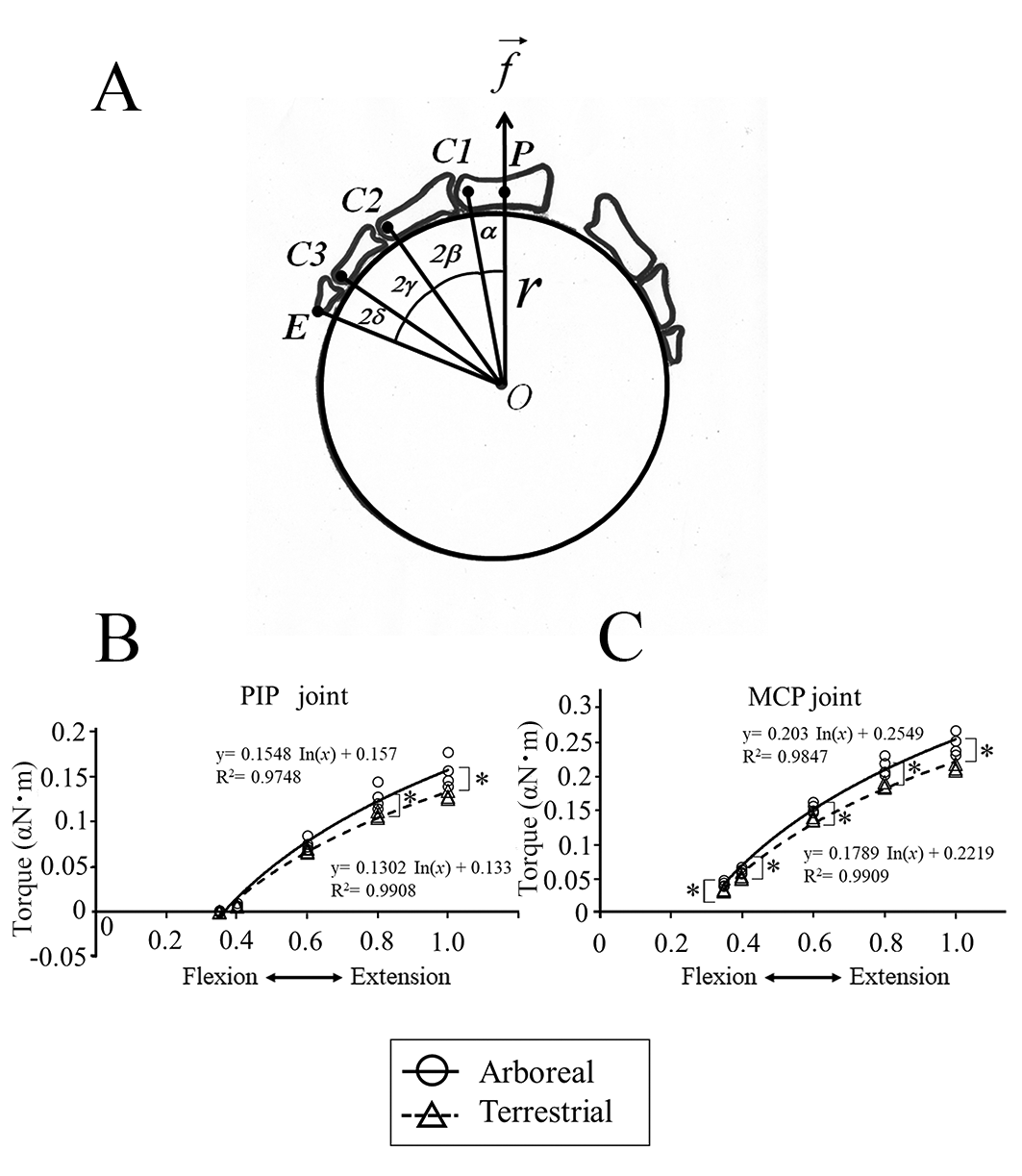

Supplement: S8 Fig — (A) A midpoint of a metacarpal bone is positioned on the top of the support. The center and radius of the cylinder and torques loaded on metacarpophalangeal (MCP) and proximal interphalangeal (PIP) joints are defined as O, r, τMCP, and τPIP, respectively. f→, the reaction force against the gravity during the arboreal quadrupedal locomotion; α, β, γ, and δ, inscribe angles of the lengths of the metacarpal bone, and proximal, middle, and distal phalanges. The torques on (B) MCP and (C) PIP joints, regression equations, and determinant coefficients (R2) were shown in arboreal (open circle) and terrestrial (open triangle) quadrupedal primates. (TIF) [file pone.0232397.s008.tif]
