# Supplementary material for: Correlation between musculoskeletal structure of the hand and primate locomotion: Morphometric and mechanical analysis in prehension using the cross- and triple-ratios
Source: PLoS One. 2020 May 4;15(5):e0232397. doi: 10.1371/journal.pone.0232397 (PMC7197777; doi:10.1371/journal.pone.0232397)
Supplement: S1 Appendix — (DOCX) [file pone.0232397.s009.docx]

**S1 Appendix**

Information about non-human primate experiment.

Hand samples of four crab-eating macaques were carefully transferred from other ophthalmic experiments, which were approved by the Institutional Review Board of Shiga University of Medical Science Animal Care and Use Committee (2012-5-5 and 2014-9-9) and conducted in this center, after the completion of those ophthalmic experiments. In those ophthalmic experiment, the temperature and humidity in the animal rooms were maintained at 25 ± 2C and 50 ± 5%, respectively. The crab eating monkeys were pair-housed in combined with two cages as followings. They were moved to individual cages before a day of the experiments. Size of each cage with a step and hanging down-bars was width 500 X depth 800 X height 800 mm. Mirror and tongs were occasionally provided to the monkeys as enrichment. The light cycle was 12 h of artificial light from 8:00 to 20:00. Each animal was fed 20 g/kg/day of commercial pellet monkey chow (CMK-1; CLEA Japan, Tokyo, Japan) with puzzle feeding trays in the morning supplemented with 20–50 g of sweet potato and half a banana in the afternoon. Water was supplied ad libitum by an automatic supplier.

Those monkeys were euthanized with 100 mg/kg sodium pentobarbital (Kyoritsu Seiyaku Corporation, Tokyo, Japan) through intravenous route, at the ophthalmic experiments.
