# Supplementary material for: Correlation between musculoskeletal structure of the hand and primate locomotion: Morphometric and mechanical analysis in prehension using the cross- and triple-ratios
Source: PLoS One. 2020 May 4;15(5):e0232397. doi: 10.1371/journal.pone.0232397 (PMC7197777; doi:10.1371/journal.pone.0232397)
Supplement: S2 Appendix — (DOCX) [file pone.0232397.s010.docx]

**S2 Appendix. Torque calculation on the joints during a power grasp**

We calculated the holding torques of CMC, MCP, PIP, and DIP joints, i.e., $\tau_{\mathrm{CMC}}$,$\tau_{\mathrm{MCP}}$,$\tau_{\mathrm{PI}P}$, and $\tau_{\mathrm{DI}P}$, respectively, as follows:

$$\tau=\left( \begin{matrix} \tau_{\mathrm{CMC}}=\tau_{1} \\ \tau_{\mathrm{MCP}}=\tau_{2} \\ \begin{matrix} \tau_{\mathrm{PIP}}=\tau_{3} \\ \tau_{\mathrm{DIP}}=\tau_{4} \end{matrix} \end{matrix} \right)=\sum_{m=1}^{4} {J^{(m)}}^{T}{{}^{0}R}_{Hm}{{}^{Hm}f}_{Hm}$$

where, $J^{(m)}$ is the Jacobian matrix and ${{}^{0}R}_{Hm}$ is the rotation matrix.

$$J^{\left( 1 \right)}=\left[ \begin{matrix} -\frac{l_{mc}}{2}S_{1} & 0 & 0 & 0 \\ {\frac{l_{mc}}{2}C}_{1} & 0 & 0 & 0 \end{matrix} \right], J^{\left( 2 \right)}=\left[ \begin{matrix} -\left( l_{mc}S_{1}+{\frac{l_{pp}}{2}S}_{12} \right) & {-\frac{l_{pp}}{2}S}_{12} & 0 & 0 \\ {l_{mc}C}_{1}+{\frac{l_{pp}}{2}C}_{12} & {\frac{l_{pp}}{2}C}_{12} & 0 & 0 \end{matrix} \right],$$

$$J^{\left( 3 \right)}=\left[ \begin{matrix} -\left( l_{mc}S_{1}+{l_{pp}S}_{12}+{\frac{l_{ip}}{2}S}_{123} \right) & -\left( {l_{pp}S}_{12}+{\frac{l_{ip}}{2}S}_{123} \right) & -{\frac{l_{ip}}{2}S}_{123} & 0 \\ {l_{mc}C}_{1}+{l_{pp}C}_{12}+{\frac{l_{ip}}{2}C}_{123} & {l_{pp}C}_{12}+{\frac{l_{ip}}{2}C}_{123} & {\frac{l_{ip}}{2}C}_{123} & 0 \end{matrix} \right],$$

$$J^{\left( 4 \right)}=\left[ \begin{matrix} -\left( l_{mc}S_{1}+{l_{pp}S}_{12}+{l_{ip}S}_{123}+{\frac{l_{dp}}{2}S}_{1234} \right) & -\left( {l_{pp}S}_{12}+{l_{ip}S}_{123}+{\frac{l_{dp}}{2}S}_{1234} \right) & -\left( {l_{ip}S}_{123}+{\frac{l_{dp}}{2}S}_{1234} \right) & {-\frac{l_{dp}}{2}S}_{1234} \\ {l_{mc}C}_{1}+{l_{pp}C}_{12}+{l_{ip}C}_{123}+{\frac{l_{dp}}{2}C}_{1234} & {l_{pp}C}_{12}+{l_{ip}C}_{123}+{\frac{l_{dp}}{2}C}_{1234} & {l_{ip}C}_{123}+{\frac{l_{dp}}{2}C}_{1234} & {\frac{l_{dp}}{2}C}_{1234} \end{matrix} \right]$$

$${{}^{0}R}_{H1}\mathbf{=}\left[ \begin{matrix} C_{1} & {-S}_{1} \\ S_{1} & C_{1} \end{matrix} \right]\boldsymbol{,}{{}^{0}R}_{H2}\mathbf{=}\left[ \begin{matrix} C_{12} & {-S}_{12} \\ S_{12} & C_{12} \end{matrix} \right]$$

$${{}^{0}R}_{H3}\mathbf{=}\left[ \begin{matrix} C_{123} & {-S}_{123} \\ S_{123} & C_{123} \end{matrix} \right]\boldsymbol{,}{{}^{0}R}_{H4}\mathbf{=}\left[ \begin{matrix} C_{1234} & {-S}_{1234} \\ S_{1234} & C_{1234} \end{matrix} \right]$$

$${{}^{H1}f}_{H1}\mathbf{=}\left[ \begin{matrix} 0 \\ f_{H1} \end{matrix} \right]\boldsymbol{,}{{}^{H2}f}_{H2}\mathbf{=}\left[ \begin{matrix} 0 \\ f_{H2} \end{matrix} \right]\boldsymbol{,}{{}^{H3}f}_{H3}\mathbf{=}\left[ \begin{matrix} 0 \\ f_{H3} \end{matrix} \right]\boldsymbol{,}{{}^{H4}f}_{H4}\mathbf{=}\left[ \begin{matrix} 0 \\ f_{H4} \end{matrix} \right]$$

The joint angles of CMC, MCP, PIP, and DIP are expressed as *θ_1_*, *θ_2,_ θ_3_*,* * and *θ_4_* (Fig 1B), and *C_klmn_* and *S_klmn_* mean $\cos\left( \theta_{k}+\theta_{l}{+\theta}_{m}{+\theta}_{n} \right)$ and $\sin\left( \theta_{k}+\theta_{l}{+\theta}_{m}{+\theta}_{n} \right)$, respectively. The torque vector is then calculated using the following formula:

$$\tau=\left( \begin{matrix} \tau_{\mathrm{CMC}} \\ \tau_{\mathrm{MCP}} \\ \begin{matrix} \tau_{\mathrm{PIP}} \\ \tau_{\mathrm{DIP}} \end{matrix} \end{matrix} \right)=\left( \begin{matrix} \begin{matrix} \frac{1}{2}\left( {l_{\mathrm{mc}}f}_{H1}+{l_{\mathrm{pp}}f}_{H2}+{l_{\mathrm{ip}}f}_{H3}+{l_{\mathrm{dp}}f}_{H4} \right)+{l_{\mathrm{mc}}f}_{H2}+{l_{\mathrm{pp}}f}_{H3C_{3}+}{l_{\mathrm{mc}}f}_{H3C_{23}}+l_{\mathrm{ip}}f_{H4}C_{4}+{l_{\mathrm{pp}}f}_{H4}C_{34}+{l_{\mathrm{mc}}f}_{H4}C_{234} \\ \frac{1}{2}\left( {l_{\mathrm{pp}}f}_{H2}+{l_{\mathrm{ip}}f}_{H3}+{l_{\mathrm{dp}}f}_{H4} \right)+{l_{\mathrm{pp}}f}_{H3}C_{3}+l_{\mathrm{ip}}f_{H4}C_{4}+{l_{\mathrm{pp}}f}_{H4}C_{34} \end{matrix} \\ \frac{1}{2}\left( {l_{\mathrm{ip}}f}_{H3}+{l_{\mathrm{dp}}f}_{H4} \right)+l_{\mathrm{ip}}f_{H4}C_{4} \\ \frac{1}{2}{l_{\mathrm{dp}}f}_{H4} \end{matrix} \right)$$

where, *l*_mc_, *l*_pp_, *l*_ip_,and *l*_dp_ are the lengths of the metacarpal bone and the distal, middle, and proximal phalanges, respectively, and the reaction forces exerted on the metacarpal region and phalanges are *f_H1_*, *f_H2_*, *f_H3_*, and *f_H4_*, respectively (Fig 1B). Based on the assumptions given above, the joint torque ($\tau_{s}$) in the simple joint model is $\frac{b^{2}\left| f \right|}{2r}$

(S2 Fig) and $\tau_{s}$ is described as $\frac{b}{2}\left| f_{H2} \right|$. Consequently, the magnitude of the reaction force exerted on the bone in the simple joint model is described as follows:

$$\left| f_{H2} \right|=\frac{b\left| f \right|}{r}=\alpha b$$

where, *r* is the radius of the object grasped in S1 Fig, *f* is the reaction force exerted on the bone from the center of the object, *b* is the bone length, and α is the proportional coefficient $\frac{\left| f \right|}{r}$. The magnitude of $f_{H2}$ is proportional to the bone length *b*, therefore, the reaction force $f_{Hm}$ is proportional to the length of the metacarpal bone and the phalanges (S2 Fig), leading to the following correlation.

$$\left( \begin{matrix} \tau_{\mathrm{CMC}} \\ \tau_{\mathrm{MCP}} \\ \begin{matrix} \tau_{\mathrm{PIP}} \\ \tau_{\mathrm{DIP}} \end{matrix} \end{matrix} \right)=\left( \begin{matrix} \begin{matrix} \frac{1}{2}\alpha\left( \left( l_{\mathrm{mc}}^{2}+l_{\mathrm{pp}}^{2}+l_{\mathrm{ip}}^{2}+l_{\mathrm{dp}}^{2} \right)+l_{\mathrm{mc}}l_{\mathrm{pp}}C_{2} +l_{\mathrm{pp}}l_{\mathrm{ip}}C_{3}+l_{\mathrm{mc}}{l_{\mathrm{ip}} C}_{23} +l_{\mathrm{ip}}{l_{\mathrm{dp}}C}_{4}+l_{\mathrm{pp}}l_{\mathrm{dp}}C_{34} +l_{\mathrm{mc}}{l_{\mathrm{dp}}C}_{234} \right) \\ \frac{1}{2}\alpha\left( \left( l_{\mathrm{pp}}^{2}+l_{\mathrm{ip}}^{2}+l_{\mathrm{dp}}^{2} \right)+l_{\mathrm{pp}} l_{\mathrm{ip}}C_{3}+l_{\mathrm{ip}}{l_{\mathrm{dp}}C}_{4}+{l_{\mathrm{pp}}l_{\mathrm{dp}}C}_{34} \right) \end{matrix} \\ \frac{1}{2}\alpha\left( \left( l_{\mathrm{ip}}^{2}+l_{\mathrm{dp}}^{2} \right)+l_{\mathrm{ip}}{l_{\mathrm{dp}}C}_{4} \right) \\ \frac{1}{2}\alpha l_{\mathrm{dp}}^{2} \end{matrix} \right)$$
