# Supplementary material for: Correlation between musculoskeletal structure of the hand and primate locomotion: Morphometric and mechanical analysis in prehension using the cross- and triple-ratios
Source: PLoS One. 2020 May 4;15(5):e0232397. doi: 10.1371/journal.pone.0232397 (PMC7197777; doi:10.1371/journal.pone.0232397)
Supplement: S3 Appendix — (DOCX) [file pone.0232397.s011.docx]

**S3 Appendix. Torque calculation on the joints during suspensory hand postures**

Since the PIP joint is positioned on the top of the support in suspensory hand postures in *Hylobates* spp. and *Ateles* spp., the torque during suspensory hand postures can be calculated according to the following equations. On a tree branch, the force on each point of the phalanx, which supports the body weight against gravity during the suspensory hand posture, has been defined as$\vec{f}$(S3 Fig). The distance between that point loaded with $\vec{f}$and the joint is $\vec{l}$ in S3 Fig.

$$\vec{l}=\left( r\sin\theta,r(\cos\theta-1 \right),0)$$

$$\vec{f}=(0,f_{u}r\cos\theta d\theta,0)$$

where, $f_{u}$ is a force per unit length.

Torque ($\left| d\tau\right|$) loaded on the infinitesimal interval $rd\theta$ is described as follows:

$$\left| d\vec{\tau} \right|=d\vec{\tau_{z}}={(\vec{l}\times\vec{f})}_{z}=l_{x}f_{y}-l_{y}f_{x}=f_{u}r^{2}\sin\theta\cos\theta d\theta=\frac{1}{2}f_{u}r^{2}\sin2\theta d\theta$$

The integral of $\left| d\vec{\tau} \right|$ from 0 to $2\gamma$ along an arc shows the net torque on the PIP joint by $\vec{f}$ loaded directly on the middle phalanx.

$$\tau_{\mathrm{PIP}}^{\#}=\frac{1}{2}f_{u}r^{2}\int_{0}^{2\gamma} \sin2\theta d\theta=-\frac{1}{2}f_{u}r^{2}\left[ \frac{1}{2}\cos2\theta\right]_{0}^{2\gamma}=-\frac{1}{4}f_{u}r^{2}(\cos4\gamma-1)$$

$$=\frac{1}{4}f_{u}r^{2}\left( 1-\cos4\gamma\right)= \frac{1}{2}f_{u}r^{2}\sin^{2} 2\gamma(10)$$

Similarly,

$$\tau_{\mathrm{MCP}}^{\#}=f_{u}r^{2}\sin2\beta\int_{\frac{\pi}{2}-2\beta}^{\frac{\pi}{2}+2\gamma} \sin\theta d\theta-\frac{1}{2}f_{u}r^{2}\int_{\frac{\pi}{2}-2\beta}^{\frac{\pi}{2}+2\gamma} \sin2\theta d\theta$$

$$= \frac{1}{2}f_{u}r^{2} {(\sin2\beta+\sin2\gamma)}^{2} (11)$$

$$\tau_{\mathrm{MCP}}=f_{u}r^{2}\sin2\beta\int_{\frac{\pi}{2}-2\beta}^{\frac{\pi}{2}+2\gamma+2\delta} \sin\theta d\theta-\frac{1}{2}f_{u}r^{2}\int_{\frac{\pi}{2}-2\beta}^{\frac{\pi}{2}+2\gamma+2\delta} \sin2\theta d\theta$$

$$= \frac{1}{2}f_{u}r^{2} {(\sin2\beta+\sin(2\gamma+2\delta))}^{2} (12)$$

$$\tau_{\mathrm{PIP}}=\frac{1}{2}f_{u}r^{2}\int_{0}^{2\gamma+2\delta} \sin2\theta d\theta$$

$$= \frac{1}{2}f_{u}r^{2}\sin^{2} (2\gamma+2\delta) (13)$$

Hence, the following equation is introduced using eqs. 10–13:

$$\sqrt{\frac{\tau_{\mathrm{MCP}}^{\#}}{\tau_{\mathrm{PIP}}^{\#}}}\cdot\sqrt{\frac{\tau_{\mathrm{PIP}}}{\tau_{\mathrm{MCP}}}}=\frac{\sin2\beta+\sin2\gamma}{sin2 \gamma}\cdot\frac{\sin\left( 2\gamma+2\delta\right)}{\sin2\beta+\sin\left( 2\gamma+2\delta\right)}$$

$$=\frac{l_{\mathrm{pp}}\cos\beta+l_{\mathrm{ip}}\cos\gamma}{l_{\mathrm{ip}}\cos\gamma}\cdot\frac{\left| \vec{l_{\mathrm{ip}}}+\vec{l_{\mathrm{dp}}} \right|\cos\left( \gamma+\delta\right)}{l_{2}\cos\beta+\left| \vec{l_{\mathrm{ip}}}+\vec{l_{\mathrm{dp}}} \right|\cos\left( \gamma+\delta\right)} (14)$$

where, $l_{n}$ is the length of the phalanx. This equation shows that the torque ratios during suspensory hand postures are calculated from the lengths of the orthogonal projection images of the phalanges to line *L*, parallel with the x-axis (S4 Fig). If $\beta,\gamma,\delta=0$, in other words, if the finger is fully extended and straightened, Eq. 14 is equal to the Ph cross-ratio, therefore, the hand cross-ratio correlates with torque distribution during brachiation.

On the other hand, the following equations are induced during suspensory hand postures (S3 Fig):

$$l_{\mathrm{pp}}=2rsin \beta,l_{\mathrm{ip}} =2rsin \gamma,\theta_{3}=\beta+\gamma(15)$$

hence,

$$arc\sin\left( \frac{l_{\mathrm{pp}}}{2r}\sqrt{1-\frac{l_{\mathrm{ip}}^{2}}{4r^{2}}}+\frac{l_{\mathrm{ip}}}{2r}\sqrt{1-\frac{l_{\mathrm{pp}}^{2}}{4r^{2}}} \right)=\left\{ \begin{aligned} \theta_{3} \\ (\pi-\theta_{3}) \end{aligned} \right.$$

$$\sin\left( \pi-\theta_{3} \right)=\sin\theta_{3}=\frac{l_{\mathrm{pp}}}{2r}\sqrt{1-\frac{l_{\mathrm{ip}}^{2}}{4r^{2}}}+\frac{l_{\mathrm{ip}}}{2r}\sqrt{1-\frac{l_{\mathrm{pp}}^{2}}{4r^{2}}}$$

$$4r^{2}\sin\theta_{3}=l_{\mathrm{pp}}\sqrt{4r^{2}-l_{\mathrm{ip}}^{2}}+l_{\mathrm{ip}}\sqrt{4r^{2}-l_{\mathrm{pp}}^{2}}$$

then, $r$ is calculated by taking the logarithm of both sides:

$$log\left( 4r^{2}\sin\theta_{3}-l_{\mathrm{pp}}\sqrt{4r^{2}-l_{\mathrm{ip}}^{2}} \right)=log\left( l_{\mathrm{ip}}\sqrt{4r^{2}-l_{\mathrm{pp}}^{2}} \right)$$

$$\left( \frac{4r^{2}\sin\theta_{3}-l_{\mathrm{pp}}\sqrt{4r^{2}-l_{\mathrm{ip}}^{2}}}{l_{\mathrm{ip}}} \right)^{2}=4r^{2}-l_{\mathrm{pp}}^{2}$$

$$r^{2}=\frac{l_{\mathrm{pp}}^{2}+l_{\mathrm{ip}}^{2}+2l_{\mathrm{pp}}l_{\mathrm{ip}}\cos\theta_{3}}{4\sin^{2} \theta_{3}} (16)$$

where, $r$ is a real number, and the range of $\theta_{3}$is from 0 to $\frac{\pi}{2}$.

Then, $\tau_{\mathrm{MCP}}^{\#}$ and $\tau_{\mathrm{PIP}}^{\#}$ during suspensory hand postures are calculated using eqs. 10–16.
