# Supplementary material for: Correlation between musculoskeletal structure of the hand and primate locomotion: Morphometric and mechanical analysis in prehension using the cross- and triple-ratios
Source: PLoS One. 2020 May 4;15(5):e0232397. doi: 10.1371/journal.pone.0232397 (PMC7197777; doi:10.1371/journal.pone.0232397)
Supplement: S4 Appendix — (DOCX) [file pone.0232397.s012.docx]

**S4 Appendix. Relationship between the cross-ratio and torque ratios between the joints**

The factors in cross- and triple-ratios are described as follows.

$$\left| \vec{l_{\mathrm{mc}}}+\vec{l_{\mathrm{pp}}}+\vec{l_{\mathrm{ip}}}+\vec{l_{\mathrm{dp}}} \right|$$

$$=\sqrt{\left( {l_{\mathrm{mc}}C}_{1}+l_{\mathrm{pp}}C_{12}+ l_{\mathrm{ip}}C_{123}+l_{\mathrm{dp}}C_{1234} \right)^{2}+\left( l_{\mathrm{mc}}S_{1}+ l_{\mathrm{pp}}S_{12}+ l_{\mathrm{ip}}S_{123}+l_{\mathrm{dp}}S_{1234} \right)^{2}}$$

$$=\sqrt{l_{\mathrm{mc}}^{2}+l_{\mathrm{pp}}^{2}+l_{\mathrm{ip}}^{2}+l_{dp}^{2}+2\left( l_{\mathrm{mc}}{l_{\mathrm{pp}}C}_{2} +l_{\mathrm{pp}}l_{\mathrm{ip}} C_{3}+l_{\mathrm{mc}} {l_{\mathrm{ip}}C}_{23} + l_{\mathrm{ip}} l_{\mathrm{dp}}C_{4}+l_{\mathrm{pp}}l_{\mathrm{dp}}C_{34} +l_{\mathrm{mc}}{l_{\mathrm{dp}}C}_{234} \right)}$$

$$=\sqrt{\frac{2\tau_{\mathrm{CMC}}}{\alpha}}$$

Similarly, the following values were calculated:

$$\left| \vec{l_{\mathrm{pp}}}+\vec{l_{\mathrm{ip}}}+\vec{l_{\mathrm{dp}}} \right|=\sqrt{l_{\mathrm{pp}}^{2}+l_{\mathrm{ip}}^{2}+l_{dp}^{2}+2\left( l_{\mathrm{pp}} {l_{\mathrm{ip}}C}_{3}+l_{\mathrm{ip}} l_{\mathrm{dp}}C_{4}+ l_{\mathrm{pp}}l_{\mathrm{dp}}C_{34} \right)}=\sqrt{\frac{2\tau_{\mathrm{MCP}}}{\alpha}}$$

$$\left| \vec{l_{\mathrm{ip}}}+\vec{l_{\mathrm{dp}}} \right|=\sqrt{l_{\mathrm{ip}}^{2}+l_{dp}^{2}+2l_{\mathrm{ip}} l_{\mathrm{dp}} C_{4}}=\sqrt{\frac{2\tau_{\mathrm{PIP}}}{\alpha}}$$

$$\left| \vec{l_{\mathrm{pp}}}+\vec{l_{\mathrm{ip}}} \right|=\sqrt{l_{\mathrm{pp}}^{2}+l_{\mathrm{ip}}^{2}+2{l_{\mathrm{pp}}l_{\mathrm{ip}}C}_{3}}=\sqrt{\frac{2\tau_{\mathrm{MCP}}^{\#}}{\alpha}}$$

$$\left| \vec{l_{\mathrm{ip}}} \right|=\sqrt{l_{\mathrm{ip}}^{2}}=\sqrt{\frac{2\tau_{\mathrm{PIP}}^{\#}}{\alpha}}$$

$$\left| \vec{l_{\mathrm{mc}}}+\vec{l_{\mathrm{pp}}} \right|=\sqrt{l_{\mathrm{mc}}^{2}+l_{\mathrm{pp}}^{2}+2{{l_{\mathrm{mc}}l}_{\mathrm{pp}}C}_{2}}=\sqrt{\frac{2\tau_{\mathrm{CMC}}^{\#\#}}{\alpha}}$$

$$\left| \vec{l_{\mathrm{pp}}} \right|=\sqrt{l_{\mathrm{pp}}^{2}}=\sqrt{\frac{2\tau_{\mathrm{MCP}}^{\#\#}}{\alpha}}$$

where, $\tau_{\mathrm{MCP}}^{\#}$ and $\tau_{\mathrm{PIP}}^{\#}$are the torques loaded on MCP and PIP joints, respectively, when the force loaded on the distal phalanx is not considered, i.e., torques on MP and PIP joints when an object is grasped without using the distal phalanx. Similarly, $\tau_{\mathrm{CMC}}^{\#\#}$and $\tau_{\mathrm{MCP}}^{\#\#}$ are the torques loaded on CMC and MCP joints, respectively, when an object is grasped without using the distal and middle phalanges.

Thus, the following equations can be derived:

$$Ph cross-ratio= \frac{\left| \vec{l_{\mathrm{pp}}}+\vec{l_{\mathrm{ip}}} \right|\left| \vec{l_{\mathrm{ip}}}+\vec{l_{\mathrm{dp}}} \right|}{\left| \vec{l_{\mathrm{ip}}} \right|\left| \vec{l_{\mathrm{pp}}}+\vec{l_{\mathrm{ip}}}+\vec{l_{\mathrm{dp}}} \right|}=\sqrt{\frac{\tau_{\mathrm{PIP}}}{\tau_{\mathrm{MCP}}}}\cdot\sqrt{\frac{\tau_{\mathrm{MCP}}^{\#}}{\tau_{\mathrm{PIP}}^{\#}}}$$

Similarly,

$$MPh cross-ratio= \frac{\left| \vec{l_{\mathrm{mc}}}+\vec{l_{\mathrm{pp}}} \right|\left| \vec{l_{\mathrm{pp}}}+\vec{l_{\mathrm{ip}}}+\vec{l_{\mathrm{dp}}} \right|}{\left| \vec{l_{\mathrm{pp}}} \right|\left| \vec{l_{\mathrm{mc}}}+\vec{l_{\mathrm{pp}}}+\vec{l_{\mathrm{ip}}}+\vec{l_{\mathrm{dp}}} \right|}=\sqrt{\frac{\tau_{\mathrm{MCP}}}{\tau_{\mathrm{CMC}}}}\cdot\sqrt{\frac{\tau_{\mathrm{CMC}}^{\#\#}}{\tau_{\mathrm{MCP}}^{\#\#}}}$$

then, using Eq. (2),

$$\mathrm{Triple}-ratio=\sqrt{\frac{\tau_{\mathrm{PIP}}}{\tau_{\mathrm{CMC}}}}\cdot\sqrt{\frac{\tau_{\mathrm{MCP}}^{\#}}{\tau_{\mathrm{PIP}}^{\#}}}\cdot\sqrt{\frac{\tau_{\mathrm{CMC}}^{\#\#}}{\tau_{\mathrm{MCP}}^{\#\#}}}$$

In suspensory hand postures, $\sqrt{\frac{\tau_{\mathrm{MCP}}^{\#}}{\tau_{\mathrm{PIP}}^{\#}}}\cdot\sqrt{\frac{\tau_{\mathrm{PIP}}}{\tau_{\mathrm{MCP}}}}$ is described as the following equation

(S3 Appendix, and S3 Fig):

$$\sqrt{\frac{\tau_{\mathrm{MCP}}^{\#}}{\tau_{\mathrm{PIP}}^{\#}}}\cdot\sqrt{\frac{\tau_{\mathrm{PIP}}}{\tau_{\mathrm{MCP}}}}=\frac{l_{\mathrm{pp}}\cos\beta+l_{\mathrm{ip}}\cos\gamma}{l_{\mathrm{ip}}\cos\gamma}\cdot\frac{\left| \vec{l_{\mathrm{ip}}}+\vec{l_{\mathrm{dp}}} \right|\cos\left( \gamma+\delta\right)}{l_{pp}\cos\beta+\left| \vec{l_{\mathrm{ip}}}+\vec{l_{\mathrm{dp}}} \right|\cos\left( \gamma+\delta\right)} (17)$$

where, *l*_pp_ , *l*_ip_ , *l*_dp_ and are the lengths of proximal, middle, and distal phalanges, respectively. The torque ratio during a suspensory hand posture is the same as the Ph cross-ratio when the finger is straightened. This suggests that the Ph cross-ratio is correlated to torque ratios in a suspensory hand posture as well as in a cylindrical grasp.
