# Supplementary material for: Correlation between musculoskeletal structure of the hand and primate locomotion: Morphometric and mechanical analysis in prehension using the cross- and triple-ratios
Source: PLoS One. 2020 May 4;15(5):e0232397. doi: 10.1371/journal.pone.0232397 (PMC7197777; doi:10.1371/journal.pone.0232397)
Supplement: S5 Appendix — (DOCX) [file pone.0232397.s013.docx]

**S5 Appendix. Calculation of torque on the joints during quadrupedal locomotion on a tree branch**

In this posture, the primary loading area is the palm; therefore, we assumed that the midpoint of the metacarpal bone is positioned on the top of the support, and the torque during quadrupedal postures can be calculated according to Eq. (10). On a tree branch, the force on each point of the phalanx, which supports the body weight against gravity, is defined as$\vec{f}$(S8 Fig). The torque on MCP and PIP joints $\left( \alpha+2\beta+2\gamma+2\delta\leq\frac{\pi}{2} \right)$ was calculated as follows:

$$\tau_{\mathrm{MCP}}= \frac{1}{2}f_{u}r^{2}\sin^{2} \left( \alpha+2\beta+2\gamma+2\beta\right)- \frac{1}{2}f_{u}r^{2}\sin^{2} \left( \alpha\right)$$

$$\tau_{\mathrm{PI}P}= \frac{1}{2}f_{u}r^{2}\sin^{2} \left( \alpha+2\beta+2\gamma+2\beta\right)- \frac{1}{2}f_{u}r^{2}\sin^{2} \left( \alpha+2\beta\right)$$

where $f_{u}$ is force per unit length.

In the case of $\alpha+2\beta+2\gamma+2\delta>\frac{\pi}{2}$, such torque is described by the following equations:

$$\tau_{\mathrm{MCP}}= \frac{1}{2}f_{u}r^{2}\sin^{2} \left( \frac{\pi}{2} \right)- \frac{1}{2}f_{u}r^{2}\sin^{2} \left( \alpha\right)$$

$$\tau_{\mathrm{PIP}}= \frac{1}{2}f_{u}r^{2}\sin^{2} \left( \frac{\pi}{2} \right)- \frac{1}{2}f_{u}r^{2}\sin^{2} \left( \alpha+2\beta\right)$$
