# Supplementary material for: Correlation between musculoskeletal structure of the hand and primate locomotion: Morphometric and mechanical analysis in prehension using the cross- and triple-ratios
Source: PLoS One. 2020 May 4;15(5):e0232397. doi: 10.1371/journal.pone.0232397 (PMC7197777; doi:10.1371/journal.pone.0232397)
Supplement: S1 Table — (DOCX) [file pone.0232397.s014.docx]

| S1 Table The information of the primates examined in this study | | | | |  |
| --- | --- | --- | --- | --- | --- |
| SID | Specises | Origin | SID | Specises | Origin |
| 1 | *Homo sapiens #1* | G | 11 | *Cercopithecus diana* | G |
|  | *Homo sapiens #2* | G | 12 | *Cercopithecus neglectus* | T |
|  | *Homo sapiens #3* | S | 13 | *Macaca cyclopis* | T |
|  | *Homo sapiens #4* | S | 14 | *Macaca fascicularis #1* | SM |
|  | *Homo sapiens #5* | S |  | *Macaca fascicularis #2* | SM |
|  | *Homo sapiens #6* | S |  | *Macaca fascicularis #3* | SM |
|  | *Homo sapiens #7* | S |  | *Macaca fascicularis #4* | SM |
|  | *Homo sapiens #8* | S | 15 | *Macaca fuscata#1* | S |
|  | *Homo sapiens #9* | T |  | *Macaca fuscata#2* | S |
| 2 | *Gorilla gorilla #1* | T |  | *Macaca fuscata#3* | T |
|  | *Gorilla gorilla #2* | K |  | *Macaca fuscata#4* | K |
|  | *Gorilla gorilla #3* | K | 16 | *Macaca mulatta #1* | T |
|  | *Gorilla gorilla #4* | K |  | *Macaca mulatta #2* | K |
| 3 | *Pan troglodytes #1* | K | 17 | *Macaca nemestrina* | G |
|  | *Pan troglodytes #2* | K | 18 | *Macaca radiata* | T |
|  | *Pan troglodytes #3* | K | 19 | *Papio anubis* | T |
|  | *Pan troglodytes #4* | K | 20 | *Papio hamadryas #1* | K |
|  | *Pan troglodytes #5* | K |  | *Papio hamadryas #2* | K |
|  | *Pan troglodytes #6* | K |  | *Papio hamadryas #3* | K |
|  | *Pan troglodytes #7* | K |  | *Papio hamadryas #4* | K |
| 4 | *Pongo abelii* | K | 21 | *Theropithecus gelada* | T |
| 5 | *Pongo pygmaeus #1* | D | 22 | *Ateles paniscus* | T |
|  | *Pongo pygmaeus #2* | K | 23 | *Ateles belzebuth* | T |
| 6 | *Hylobates agillis* | K | 24 | *Ateles geoffroyi* | K |
| 7 | *Hylobates lar #1* | K | 25 | *Ateles sp. (unclassified)* | K |
|  | *Hylobates lar #2* | K | 26 | *Cebus capucinus* | G |
| 8 | *Hylobates pileatus* | K | 27 | *Saimiri sciureus* | T |
| 9 | *Symphalangus syndactylus* | K |  |  |  |
| 10 | *Hylobates* sp. *(unclassified) #1* | K |  |  |  |
|  | *Hylobates* sp. *(unclassified) #2* | K |  |  |  |

SID=species identification number G=Gothenburg Museum of Natural History

D=Natural History Museum of Denmark T=National Museum of Nature and Science, Tokyo

K= Primate Research Institute Kyoto University S= Shimane University

SM=Shiga University of Medical Science
