# Supplementary material for: Correlation between musculoskeletal structure of the hand and primate locomotion: Morphometric and mechanical analysis in prehension using the cross- and triple-ratios
Source: PLoS One. 2020 May 4;15(5):e0232397. doi: 10.1371/journal.pone.0232397 (PMC7197777; doi:10.1371/journal.pone.0232397)
Supplement: S2 Table — (DOCX) [file pone.0232397.s015.docx]

| S2 Table Abbreviation table | |
| --- | --- |
| **Abbreviation** | **Description** |
| *l*_dp_ | Lengths of distal phalanx |
| *l*_ip_ | Lengths of middle phalanx |
| *l*_pp_ | Lengths of proximal phalanx |
| *l*_mc_ | Lengths of metacarpal bone |
| *τ*_DIP_ | Holding torques of the distal interphalangeal joint |
| *τ*_PIP_ | Holding torques of the proximal interphalangeal joint |
| *τ*_MCP_ | Holding torques of the metacarpophalangeal joint |
| *τ*_CMC_ | Holding torques of the carpometacarpal joint |
| *τ*_DIP_*__N_* | Normalized holding torques of the distal interphalangeal joint |
| *τ*_PIP_*__N_* | Normalized holding torques of the proximal interphalangeal joint |
| *τ*_MCP_*__N_* | Normalized holding torques of the metacarpophalangeal joint |
| *τ_n_*^＃^ | Torque on the PIP,MCP,and CMC joints without contribution of the distal phalanx |
| *θ*_4_ | DIP joint angle |
| *θ*_3_ | PIP joint angle |
| *θ*_2_ | MCP joint angle |
| *θ*_1_ | CMC joint angle |
| *F_m_* | Traction force of the tendon by muscle contraction |
| *F_m_*_DIP_ | The traction force of the tendon by muscle contraction at the DIP joint |
| *F_m_*_PIP_ | The traction force of the tendon by muscle contraction at the PIP joint |
| *F_m_*_MCP_ | The traction force of the tendon by muscle contraction at the MCP joint |
| $F_{mMCP\_P}$ | The traction force of the tendon by flexor digitorum profundus muscle contraction at the MCP joint |
| $F_{mMCP\_S}$ | The traction force of the tendon by flexor digitorum superficialis muscle contraction at the MCP joint |
| $F_{mMCP\_I}$ | The traction force of the tendon by interosseous muscle contraction at the MCP joint |
| *F_m_*[*n*]^＃^ | Indicates the traction force during suspensory hand posture |
| *lm* | Moment arm of the flexor digitorum muscle tendons |
| *f*_Hm_ | Resultant force |
| *C_klmn_* | $\cos\left( \theta_{k}+\theta_{l}{+\theta}_{m}{+\theta}_{n} \right)$ |
| *S_klmn_* | $\sin\left( \theta_{k}+\theta_{l}{+\theta}_{m}{+\theta}_{n} \right)$ |
| $f_{u}$ | Force per unit length |
| C_cyl_ | A central axis of a cylinder |
| $L$ | Total length of the phalanges ($L=l[pp]+l[ip]+ l[dp])$ |
| *f_H1_* | Reaction forces from the cylindrical object to metacarpal bone |
| *f_H2_* | Reaction forces from the cylindrical object to proximal phalanx |
| *f_H3_* | Reaction forces from the cylindrical object to middle phalanx |
| *f_H4_* | Reaction forces from the cylindrical object to distal phalanx |
| C_cyl_ | A central axis of a cylinder |
| *lm* | Moment arm of the flexor digitorum muscle tendons |
