# Supplementary material for: Correlation between musculoskeletal structure of the hand and primate locomotion: Morphometric and mechanical analysis in prehension using the cross- and triple-ratios
Source: PLoS One. 2020 May 4;15(5):e0232397. doi: 10.1371/journal.pone.0232397 (PMC7197777; doi:10.1371/journal.pone.0232397)
Supplement: S3 Table — (DOCX) [file pone.0232397.s016.docx]

|  | ***Papio hamadryas*** | ***Hylobates* spp.** | ***Ateles* sp.** |
| --- | --- | --- | --- |
| Effective echo time (ms) | 10.828 | 12.364 | 11.34 |
| Repetition times (ms) | 1600 | 1600 | 1600 |
| Slice thickness (mm) | 1 | 1 | 1 |
| Matrix (mm^2^) | 480 × 240 | 520× 360/600 × 360 | 520 × 360 |
| Field of view (mm^2^) | 120 × 60 | 130 × 90/150 × 90 | 130 × 90 |
| Resolution (mm^2^) | 0.25 × 0.25 | 0.25 × 0.25 | 0.25 × 0.25 |
| Slice gap (mm) | -0.5 | -0.5 | -0.5 |

S3 Table Parameters of MRI sequences
