# Supplementary material for: Correlation between musculoskeletal structure of the hand and primate locomotion: Morphometric and mechanical analysis in prehension using the cross- and triple-ratios
Source: PLoS One. 2020 May 4;15(5):e0232397. doi: 10.1371/journal.pone.0232397 (PMC7197777; doi:10.1371/journal.pone.0232397)
Supplement: S4 Table — (DOCX) [file pone.0232397.s017.docx]

| **DIP** | **digit Ⅱ** | **digit Ⅲ** | **digit Ⅳ** | **digit Ⅴ** |
| --- | --- | --- | --- | --- |
| *Hylobates* spp. | 7.754×10^-5^x+3.119×10^-2^ | 6.378×10^-5^x+2.954×10^-2^ | -3.325×10^-5^x+3.182×10^-2^ | -4.002×10^-5^x+3.241×10^-2^ |
| Adjusted R-Squared | -0.03947 | -0.03526 | -0.06986 | -0.06683 |
| *Papio hamadryas* | 7.836×10^-5^x+4.399×10^-2^ | 9.696×10^-5^x+4.355e-2 | 3.044×10^-5^x+4.198×10^-2^ | 4.683×10^-5^x+4.277×10^-2^ |
| Adjusted R-Squared | -0.05907 | -0.06282 | -0.07341 | -0.06985 |
| *Ateles* sp. | -3.279×10^-5^x+3.448×10^-2^ | 2.025×10^-5^x+3.236×10^-2^ | -1.638×10^-4^x+3.811×10^-2^ | 9.983×10^-6^x+3.240×10^-2^ |
| Adjusted R-Squared | -0.3148 | -0.3287 | -0.2657 | -0.3330 |
|  |  |  |  |  |
| **PIP** | **digit Ⅱ** | **digit Ⅲ** | **digit Ⅳ** | **digit Ⅴ** |
| *Hylobates* spp. | 9.489×10^-5^x+5.841×10^-2^ | 1.458×10^-4^x+5.523×10^-2^ | 7.263×10^-5^x+5.711×10^-2^ | 2.111×10^-4^x+6.294×10^-2^ |
| Adjusted R-Squared | 0.06907 | 0.1405 | -0.01505 | 0.3511 |
| *Papio hamadryas* | 3.826×10^-6^x+9.867×10^-2^ | 4.670×10^-6^x+8.301×10^-2^ | 2.620×10^-6^x+8.853×10^-2^ | 4.566×10^-6^x+8.864×10^-2^ |
| Adjusted R-Squared | 0.221 | 0.4367 | 0.1429 | 0.2339 |
| *Ateles* sp. | 9.611×10^-5^x+8.224×10^-2^ | 1.294×10^-4^x+7.259×10^-2^ | 1.139×10^-5^x+7.900×10^-2^ | 1.544×10^-4^x+7.906×10^-2^ |
| Adjusted R-Squared | 0.1936 | 0.2789 | -0.2437 | 0.5366 |

S4 Table Regression equations of the normalized moment arm on the finger joint angle during a cylindrical grip

| **FDP** | **digit Ⅱ** | **digit Ⅲ** | **digit Ⅳ** | **digit Ⅴ** |
| --- | --- | --- | --- | --- |
| *Hylobates* spp. | 9.191×10^-5^x+7.654×10^-2^ | 1.139×10^-4^x+6.525×10^-2^ | 1.534×10^-4^x+6.519×10^-2^ | 2.550×10^-4^x+6.753×10^-2^ |
| Adjusted R-Squared | 0.2099 | 0.2516 | 0.6466 | 0.5329 |
| *Papio hamadryas* | -1.035×10^-4^x+1.507×10^-1^ | 1.923×10^-4^x+1.201e-1 | 4.021×10^-4^x+1.159×10^-1^ | 7.130×10^-4^x+1.227×10^-1^ |
| Adjusted R-Squared | -0.06189 | 0.1435 | 0.4777 | 0.3771 |
| *Ateles* sp. | 5.213×10^-4^x+6.969×10^-2^ | 3.125×10^-4^x+7.014×10^-2^ | 3.086×10^-4^x+7.054×10^-2^ | 2.666×10^-4^x+7.282×10^-2^ |
| Adjusted R-Squared | 0.8779 | 0.6306 | 0.9782 | 0.9447 |
|  |  |  |  |  |
| **FDS** | **digit Ⅱ** | **digit Ⅲ** | **digit Ⅳ** | **digit Ⅴ** |
| *Hylobates* spp. | 1.917×10^-4^x+8.574×10^-2^ | 1.705×10^-4^x+7.716×10^-2^ | 2.211×10^-4^x+7.578×10^-2^ | 3.399×10^-4^x+7.825×10^-2^ |
| Adjusted R-Squared | 0.2471 | 0.1995 | 0.4383 | 0.5746 |
| *Papio hamadryas* | -1.735×10^-5^x+1.701e-1 | 2.028×10^-4^x+1.449×10^-1^ | 4.358×10^-4^x+1.416×10^-1^ | 5.108×10^-4^x+1.485×10^-1^ |
| Adjusted R-Squared | -0.07656 | 0.07602 | 0.3401 | 0.2105 |
| *Ateles* sp. | 5.106×10^-4^x+8.545×10^-2^ | 3.821×10^-4^x+8.696×10^-2^ | 3.758×10^-4^x+8.493×10^-2^ | 2.889×10^-4^x+8.506×10^-2^ |
| Adjusted R-Squared | 0.7510 | 0.7888 | 0.9692 | 0.7838 |

| **INT** | **digit Ⅱ** | **digit Ⅲ** | **digit Ⅳ** | **digit Ⅴ** |
| --- | --- | --- | --- | --- |
| *Hylobates* spp. | 4.314×10^-6^x+6.246e×10^-2^ | -2.149×10^-6^x+5.772×10^-2^ | 8.356×10^-5^x+5.282×10^-2^ | 4.081×10^-5^x+5.873×10^-2^ |
| Adjusted R-Squared | -0.07642 | -0.07667 | 0.1594 | -0.05091 |
| *Papio hamadryas* | -1.774×10^-4^x+1.255×10^-1^ | -2.121×10^-4^x+1.069×10^-1^ | 2.639×10^-4^x+9.449×10^-2^ | -1.123×10^-4^x+1.124×10^-1^ |
| Adjusted R-Squared | -0.03639 | -0.01246 | 0.07108 | -0.04511 |
| *Ateles* sp. | 3.741×10^-4^x+5.590×10^-2^ | 1.953×10^-4^x+6.106×10^-2^ | -1.529×10^-5^x+6.372×10^-2^ | -5.075×10^-5^x+6.398×10^-2^ |
| Adjusted R-Squared | 0.8757 | 0.3020 | -0.3137 | -0.1999 |
