# Supplementary material for: Correlation between musculoskeletal structure of the hand and primate locomotion: Morphometric and mechanical analysis in prehension using the cross- and triple-ratios
Source: PLoS One. 2020 May 4;15(5):e0232397. doi: 10.1371/journal.pone.0232397 (PMC7197777; doi:10.1371/journal.pone.0232397)
Supplement: S5 Table — (DOCX) [file pone.0232397.s018.docx]

S5 Table Regression equations of the torque and torque ratio on the finger joint angle during a cylindrical grip

| $\sqrt{\frac{\tau_{\mathrm{PIP}}}{\tau_{\mathrm{MCP}}}}$ | **digit Ⅱ** | **digit Ⅲ** | **digit Ⅳ** | **digit Ⅴ** |
| --- | --- | --- | --- | --- |
| *Hylobates* spp. | 2.735×10^-5^x^2^-7.362×10^-4^x+0.5334 | 1.499×10^-5^x^2^+6.837×10^-4^x+0.5179 | 2.326×10^-5^x^2^-4.060×10^-4^x+0.5632 | 2.736×10^-5^x^2^-1.121×10^-3^x+0.5939 |
| Adjusted R-squared | 0.9646 | 0.9438 | 0.9394 | 0.9284 |
| *Papio hamadryas* | 3.856×10^-5^x^2^-2.336×10^-3^x+0.5652 | 2.139×10^-5^x^2^-6.112×10^-4^x+0.5467 | 3.478×10^-5^x^2^-1.777×10^-3^x+0.5613 | 1.789×10^-6^x^2^+6.058×10^-4^x+0.5202 |
| Adjusted R-squared | 0.7239 | 0.7111 | 0.846 | -0.03071 |
| *Ateles* sp. | 2.934×10^-5^x^2^-1.852×10^-3^x+0.5716 | 3.914×10^-5^x^2^-2.556×10^-3^x+0.5936 | 3.831×10^-5^x^2^-2.255×10^-3^x+0.6027 | 3.773×10^-5^x^2^-2.526×10^-3^x+0.6027 |
| Adjusted R-squared | 0.9859 | 0.9973 | 0.9988 | 0.999 |
| GEE (*p*-value) | < 0.001 | < 0.01 | < 0.001 | < 0.001 |

| $\boldsymbol{\tau}_{\mathbf{DIP}}$ | **digit Ⅱ** | **digit Ⅲ** | **digit Ⅳ** | **digit Ⅴ** |
| --- | --- | --- | --- | --- |
| *Hylobates* spp. | 1.009×10^-4^x+1.024×10^-2^ | 6.814×10^-5^x+9.748×10^-3^ | 1.984×10^-5^x+1.304×10^-2^ | -7.985×10^-5^x+1.849×10^-2^ |
| Adjusted R-squared | 0.1043 | 0.1047 | -0.06335 | 0.09351 |
| *Papio hamadryas* | 2.189×10^-4^x+2.197×10^-2^ | -4.573×10^-5^x+2.342×10^-2^ | 4.227×10^-5^x+2.159×10^-2^ | 3.768×10^-5^x+2.385×10^-2^ |
| Adjusted R-squared | 0.1445 | -0.05547 | -0.06405 | -0.0683 |
| *Ateles* sp. | -1.494×10^-4^x+1.803×10^-2^ | -6.313×10^-5^x+1.643×10^-2^ | -2.918×10^-5^x+1.555×10^-2^ | 1.809×10^-5^x+1.257×10^-2^ |
| Adjusted R-squared | -0.06553 | -0.1794 | -0.3029 | -0.2787 |
| GEE (*p*-value) | < 0.001 | < 0.001 | < 0.001 | < 0.001 |

| $\boldsymbol{\tau}_{\mathbf{PIP}}$ | **digit Ⅱ** | **digit Ⅲ** | **digit Ⅳ** | **digit Ⅴ** |
| --- | --- | --- | --- | --- |
| *Hylobates* spp. | 9.535×10^-5^x+8.713×10^-2^ | 7.260×10^-5^x+9.356×10^-2^ | -2.756×10^-5^x+0.1047 | -1.851×10^-4^x+0.1138 |
| Adjusted R-squared | 0.1375 | 0.09102 | -0.05915 | 0.4381 |
| *Papio hamadryas* | -1.497×10^-4^x+9.418×10^-2^ | -1.104×10^-4^x+9.855×10^-2^ | -1.300×10^-4^x+9.780×10^-2^ | -1.057×10^-4^x+9.471×10^-2^ |
| Adjusted R-squared | 0.1418 | 0.1162 | 0.3452 | -0.04259 |
| *Ateles* sp. | -1.427×10^-4^x+9.647×10^-2^ | 1.607×10^-5^x+9.288×10^-2^ | 3.749×10^-6^x+9.818×10^-2^ | -7.931×10^-5^x+9.881×10^-2^ |
| Adjusted R-squared | 0.8162 | -0.2919 | -0.3306 | 0.4749 |
| GEE (*p*-value) | < 0.001 | < 0.01 | < 0.001 | < 0.001 |
|  |  |  |  |  |
| $\boldsymbol{\tau}_{\mathbf{MCP}}$ | **digit Ⅱ** | **digit Ⅲ** | **digit Ⅳ** | **digit Ⅴ** |
| *Hylobates* spp. | -1.394×10^-3^x+0.3080 | -1.413×10^-3^x+0.3032 | -1.743×10^-3^x+0.3082 | -2.144×10^-3^x+0.3198 |
| Adjusted R-squared | 0.5482 | 0.5023 | 0.5047 | 0.542 |
| *Papio hamadryas* | -2.164×10^-3^x+0.3289 | -2.431×10^-3^x+0.3447 | -1.651×10^-3^x+0.3302 | -1.013×10^-3^x+0.3255 |
| Adjusted R-squared | 0.4530 | 0.7566 | 0.5924 | 0.4862 |
| *Ateles* sp. | -2.371×10^-3^x+0.3324 | -3.046×10^-3^x+0.3343 | -3.451×10^-3^x+0.3334 | -3.317×10^-3^x+0.3382 |
| Adjusted R-squared | 0.967 | 0.9097 | 0.9274 | 0.7587 |
| GEE (*p*-value) | 8.847×10^-1^ | 1.282×10^-1^ | 0.08564 | < 0.05 |
