# Supplementary material for: Correlation between musculoskeletal structure of the hand and primate locomotion: Morphometric and mechanical analysis in prehension using the cross- and triple-ratios
Source: PLoS One. 2020 May 4;15(5):e0232397. doi: 10.1371/journal.pone.0232397 (PMC7197777; doi:10.1371/journal.pone.0232397)
Supplement: S6 Table — (DOCX) [file pone.0232397.s019.docx]

S6 Table Regression equations of the torque and torque ratio on the finger joint angle during a suspensory hand posture

| $\sqrt{\frac{\tau_{\mathrm{MCP}}^{\#}}{\tau_{\mathrm{PIP}}^{\#}}}$ | **digit Ⅱ** | **digit Ⅲ** | **digit Ⅳ** | **digit Ⅴ** |
| --- | --- | --- | --- | --- |
| *Hylobates* spp. | -9.683×10^-5^x^2^+3.314×10^-3^x+2.482 | -9.153×10^-5^x^2^+5.090×10^-3^x+2.281 | -1.113×10^-4^x^2^+8.437×10^-3^x+2.137 | -1.038×10^-4^x^2^+7.357×10^-3^x+2.186 |
| Adjusted R-squared | 0.9675 | 0.9748 | 0.9473 | 0.9229 |
| *Papio hamadryas* | -2.036×10^-4^x^2^+9.795×10^-3^x+2.7602 | -1.648×10^-4^x^2^+9.093×10^-3^x+2.518 | -1.332×10^-4^x^2^+6.162×10^-3^x+2.601 | -2.405×10^-4^x^2^+1.499×10^-2^x+2.5112 |
| Adjusted R-squared | 0.5779 | 0.9165 | 0.9305 | 0.2834 |
| *Ateles* sp. | -1.754×10^-4^x^2^+1.22×10^-2^x+2.323 | -1.387×10^-4^x^2^+8.815×10^-3^x+2.341 | -1.141×10^-4^x^2^+7.500×10^-3^+2.250 | -1.487×10^-4^x^2^+1.133×10^-2^x+2.184 |
| Adjusted R-squared | 0.9999 | 0.9929 | 0.9992 | 0.9864 |
| GEE (*p*-value) | < 0.001 | < 0.001 | < 0.001 | < 0.001 |

| $\boldsymbol{\tau}_{\mathbf{PIP}}^{\boldsymbol{\#}}$ | **digit Ⅱ** | **digit Ⅲ** | **digit Ⅳ** | **digit Ⅴ** |
| --- | --- | --- | --- | --- |
| *Hylobates* spp. | 1.464×10^-5^x+1.319×10^-2^ | -2.014×10^-6^x+1.595×10^-2^ | -1.722×10^-5^x+1.689×10^-2^ | -1.868×10^-5^x+1.574×10^-2^ |
| Adjusted R-squared | 0.5548 | -0.07262 | 0.1527 | 0.321 |
| *Papio hamadryas* | -2.812×10^-6^x+8.582×10^-6^ | -1.613×10^-5^x+1.111×10^-2^ | -2.284×10^-5^x+1.122×10^-2^ | -2.323×10^-5^x+1.029×10^-2^ |
| Adjusted R-squared | -0.0758 | 0.3976 | 0.3509 | 0.05673 |
| *Ateles* sp. | -1.594×10^-5^x+1.298×10^-2^ | 1.185×10^-5^x+1.230×10^-2^ | 4.496×10^-7^x+1.445×10^-2^ | -7.469×10^-6^x+1.455×10^-2^ |
| Adjusted R-squared | 0.8207 | -0.04398 | -0.3264 | -0.1195 |
| GEE (*p*-value) | < 0.001 | < 0.001 | < 0.001 | < 0.001 |

| $\boldsymbol{\tau}_{\mathbf{MCP}}^{\boldsymbol{\#}}$ | **digit Ⅱ** | **digit Ⅲ** | **digit Ⅳ** | **digit Ⅴ** |
| --- | --- | --- | --- | --- |
| *Hylobates* spp. | -2.754×10^-3^x+0.4114 | -2.967×10^-3^x+0.4343 | -2.888×10^-3^x+0.4227 | -2.393×10^-3^x+0.3812 |
| Adjusted R-squared | 0.9626 | 0.9924 | 0.9941 | 0.9599 |
| *Papio hamadryas* | -2.391×10^-3^x+0.3458 | -2.2785×10^-3^x+0.3505 | -2.331×10^-3^x+0.3555 | -2.263×10^-3^x+0.3468 |
| Adjusted R-squared | 0.8120 | 0.9579 | 0.8814 | 0.8139 |
| *Ateles* sp. | -2.947×10^-3^x+0.4091 | -2.853×10^-3^x+0.4073 | -2.880×10^-3^x+0.4161 | -2.972×10^-3^x+0.4256 |
| Adjusted R-squared | 0.9886 | 0.9984 | 0.9921 | 0.9969 |
| GEE (*p*-value) | < 0.001 | < 0.001 | < 0.001 | < 0.001 |
|  |  |  |  |  |
