# Supplementary material for: Correlation between musculoskeletal structure of the hand and primate locomotion: Morphometric and mechanical analysis in prehension using the cross- and triple-ratios
Source: PLoS One. 2020 May 4;15(5):e0232397. doi: 10.1371/journal.pone.0232397 (PMC7197777; doi:10.1371/journal.pone.0232397)
Supplement: S7 Table — (DOCX) [file pone.0232397.s020.docx]

S7 Table　Regression equations of the traction force of the flexor tendons on the finger joint angle during a cylindrical grip

| $\boldsymbol{F}_{\boldsymbol{m}\mathbf{DIP}}$ | **digit Ⅱ** | **digit Ⅲ** | **digit Ⅳ** | **digit Ⅴ** |
| --- | --- | --- | --- | --- |
| *Hylobates* spp. | 2.801×10^-3^x+0.3188 | 1.371×10^-3^x+0.3353 | 9.943×10^-4^x+0.4145 | 1.410×10^-3^x+0.5683 |
| Adjusted R-Squared | 0.02301 | -0.002162 | -0.05524 | -0.04778 |
| *Papio hamadryas* | 4.066×10^-3^x+0.5040 | -1.269×10^-3^x+0.5304 | 9.631×10^-4^x+0.5135 | 1.317×10^-4^x+0.5626 |
| Adjusted R-Squared | 0.05833 | -0.06384 | -0.07098 | -0.0767 |
| *Ateles* sp. | -4.196×10^-3^x+0.5288 | -2.089×10^-3^x+0.5055 | 1.335×10^-3^x+0.4043 | 4.378×10^-4^x+0.3907 |
| Adjusted R-Squared | -0.07298 | -0.2817 | -0.3103 | -0.3306 |
| GEE (*p*-value) | < 0.001 | < 0.001 | < 0.001 | 0.5745 |
|  |  |  |  |  |
| $\boldsymbol{F}_{\boldsymbol{m}\mathbf{PIP}}$ | **digit Ⅱ** | **digit Ⅲ** | **digit Ⅳ** | **digit Ⅴ** |
| *Hylobates* spp. | -9.791×10^-4^x+1.5255 | -2.427×10^-3^x+1.6955 | -2.339×10^-3^x+1.8458 | 6.824×10^-3^x+1.7811 |
| Adjusted R-Squared | -0.06269 | 0.03213 | -0.02736 | 0.6285 |
| *Papio hamadryas* | -3.973×10^-3^x+1.0005 | -5.293×10^-3^x+1.2445 | -3.819×10^-3^x+1.1519 | -5.100×10^-3^x+1.1511 |
| Adjusted R-Squared | 0.3571 | 0.5431 | 0.3496 | 0.1583 |
| *Ateles* sp. | -2.727×10^-3^x+1.1651 | -1.759×10^-2^x+1.2787 | -1.452×10^-4^x+1.2443 | -2.718×10^-3^x+1.2304 |
| Adjusted R-Squared | 0.5364 | 0.1055 | -0.3232 | 0.8372 |
| GEE (*p*-value) | < 0.001 | < 0.001 | < 0.001 | < 0.001 |

| $\boldsymbol{F}_{\boldsymbol{m}\mathbf{MCP}}$**（FDP）** | **digit Ⅱ** | **digit Ⅲ** | **digit Ⅳ** | **digit Ⅴ** |
| --- | --- | --- | --- | --- |
| *Hylobates* spp. | -2.082×10^-2^x+3.9975 | -2.562×10^-2^x+4.5898 | -3.235×10^-2^x+4.6717 | -4.185×10^-2^x+4.6836 |
| Adjusted R-Squared | 0.5884 | 0.573 | 0.5977 | 0.7144 |
| *Papio hamadryas* | -1.349×10^-2^x+2.1956 | -2.342×10^-2^x+2.8666 | -2.080×10^-2^x+2.8286 | -1.777×10^-2^x+2.6035 |
| Adjusted R-Squared | 0.2976 | 0.7199 | 0.661 | 0.4984 |
| *Ateles* sp. | -4.682×10^-2^x+4.5634 | -5.085×10^-2^x+4.6334 | -5.688×10^-2^x+4.6250 | -5.330×10^-2^x+4.5683 |
| Adjusted R-Squared | 0.9459 | 0.9396 | 0.9587 | 0.8603 |
| GEE (*p*-value) | < 0.001 | < 0.001 | < 0.001 | < 0.001 |
|  |  |  |  |  |
| $\boldsymbol{F}_{\boldsymbol{m}\mathbf{MCP}}$**（FDS）** | **digit Ⅱ** | **digit Ⅲ** | **digit Ⅳ** | **digit Ⅴ** |
| *Hylobates* spp. | -2.127×10^-2^x+3.6012 | -2.345×10^-2^x+3.9325 | -2.939×10^-2^x+4.0435 | -3.717×10^-2^x+4.0367 |
| Adjusted R-Squared | 0.4991 | 0.4539 | 0.519 | 0.6993 |
| *Papio hamadryas* | -1.266×10^-2^x+1.9391 | -1.893×10^-2^x+2.3724 | -1.639×10^-2^x+2.3160 | -1.213×10^-2^x+2.1768 |
| Adjusted R-Squared | 0.376 | 0.7086 | 0.6585 | 0.4707 |
| *Ateles* sp. | -3.640×10^-2^x+3.7483 | -4.124×10^-2^x+3.7459 | -4.739×10^-2^x+3.8423 | -4.501×10^-2^x+3.9145 |
| Adjusted R-Squared | 0.9417 | 0.9518 | 0.9676 | 0.7987 |
| GEE (*p*-value) | < 0.001 | < 0.001 | < 0.001 | < 0.001 |

| $\boldsymbol{F}_{\boldsymbol{m}\mathbf{MCP}}$**（INT）** | **digit Ⅱ** | **digit Ⅲ** | **digit Ⅳ** | **digit Ⅴ** |
| --- | --- | --- | --- | --- |
| *Hylobates* spp. | -2.127×10^-2^x+3.6012 | 2.392×10^-2^x+5.2431 | -3.765×10^-2^x+5.7933 | -3.927×10^-2^x+5.4807 |
| Adjusted R-Squared | 0.4991 | 0.4919 | 0.5457 | 0.382 |
| *Papio hamadryas* | -1.409×10^-2^x+2.6247 | -1.872×10^-2^x+3.2777 | -2.547×10^-2^x+3.5276 | -6.564×10^-3^x+2.9141 |
| Adjusted R-Squared | 0.4222 | 0.3262 | 0.4679 | 0.04672 |
| *Ateles* sp. | -5.785×10^-2^x+5.7394 | -5.694×10^-2^x+5.3805 | -5.371×10^-2^x+5.2441 | -4.908×10^-2^x+5.2950 |
| Adjusted R-Squared | 0.9323 | 0.9537 | 0.9284 | 0.687 |
| GEE (*p*-value) | 8.118×10^-10^ | 8.952×10^-37^ | 4.694×10^-11^ | 3.846×10^-7^ |
