# Supplementary material for: Correlation between musculoskeletal structure of the hand and primate locomotion: Morphometric and mechanical analysis in prehension using the cross- and triple-ratios
Source: PLoS One. 2020 May 4;15(5):e0232397. doi: 10.1371/journal.pone.0232397 (PMC7197777; doi:10.1371/journal.pone.0232397)
Supplement: S8 Table — (DOCX) [file pone.0232397.s021.docx]

S8 Table Regression equations of the traction force of the flexor tendon on the PIP joint angle during a suspensory hand posture

| ${\boldsymbol{F}_{\boldsymbol{m}\mathbf{PIP}}}^{\boldsymbol{\#}}$ | **digit Ⅱ** | **digit Ⅲ** | **digit Ⅳ** | **digit Ⅴ** |
| --- | --- | --- | --- | --- |
| *Hylobates* spp. | -1.3×10^-4^x+0.2295 | -6.1×10^-4^x+0.2875 | -5.7×10^-4^x+0.2969 | -8.6×10^-4^x+0.2471 |
| Adjusted R-Squared | -0.06571 | 0.1365 | 0.04887 | 0.4475 |
| *Papio hamadryas* | -2.6×10^-4^x+0.09093 | -6.3×10^-4^x+0.1402 | -5.0×10^-4^x+0.1313 | -6.6×10^-4^x+0.1244 |
| Adjusted R-Squared | 0.02999 | 0.5952 | 0.4429 | 0.1985 |
| *Ateles* sp. | -3.3×10^-4^x+0.1569 | -1.3×10^-4^x+0.1706 | -2.2×10^-5^x+0.1831 | -3.6×10^-4^x+0.1814 |
| Adjusted R-Squared | 0.7248 | -0.213 | -0.3063 | 0.7786 |
| GEE (*p*-value) | < 0.001 | < 0.001 | < 0.001 | < 0.001 |
|  |  |  |  |  |
| ${\boldsymbol{F}_{\boldsymbol{m}\mathbf{MCP}}}^{\boldsymbol{\#}}$**（FDP）** | **digit Ⅱ** | **digit Ⅲ** | **digit Ⅳ** | **digit Ⅴ** |
| *Hylobates* spp. | -3.7×10^-2^x+5.3540 | -4.6×10^-2^x+6.5049 | -4.6×10^-2^x+6.4074 | -3.9×10^-2^x+5.5812 |
| Adjusted R-Squared | 0.9535 | 0.9599 | 0.9852 | 0.9417 |
| *Papio hamadryas* | -1.7×10^-2^x+2.3581 | -2.1×10^-2^x+2.9655 | -2.4×10^-2^x+3.0905 | -2.0×10^-2^x+2.6273 |
| Adjusted R-Squared | 0.7517 | 0.9511 | 0.9395 | 0.554 |
| *Ateles* sp. | -4.8×10^-2^x+5.7643 | -4.3×10^-2^x+5.6646 | -4.3×10^-2^x+5.8099 | -4.3×10^-2^x+5.7392 |
| Adjusted R-Squared | 0.9937 | 0.9847 | 0.9965 | 0.9957 |
| GEE (*p*-value) | < 0.001 | < 0.001 | < 0.001 | < 0.001 |

| ${\boldsymbol{F}_{\boldsymbol{m}\mathbf{MCP}}}^{\boldsymbol{\#}}$**（FDS）** | **digit Ⅱ** | **digit Ⅲ** | **digit Ⅳ** | **digit Ⅴ** |
| --- | --- | --- | --- | --- |
| *Hylobates* spp. | -3.7×10^-2^x+4.9301 | -4.3×10^-2^x+5.7438 | -4.3×10^-2^x+5.6809 | -3.5×10^-2^x+4.8402 |
| Adjusted R-Squared | 0.867 | 0.8993 | 0.9616 | 0.9452 |
| *Papio hamadryas* | -1.5×10^-2^x+2.0663 | -1.7×10^-2^x+2.4168 | -1.9×10^-2^x+2.5014 | -1.5×10^-2^x+2.2043 |
| Adjusted R-Squared | 0.7892 | 0.9504 | 0.9226 | 0.6347 |
| *Ateles* sp. | -3.8×10^-2^x+4.6998 | -3.5×10^-2^x+4.5839 | -3.611×10^-2^x+4.8227 | -3.7×10^-2^x+4.9481 |
| Adjusted R-Squared | 0.9856 | 0.99 | 0.9953 | 0.9959 |
| GEE (*p*-value) | < 0.001 | < 0.001 | < 0.001 | < 0.001 |
|  |  |  |  |  |
| ${\boldsymbol{F}_{\boldsymbol{m}\mathbf{MCP}}}^{\boldsymbol{\#}}$**（INT）** | **digit Ⅱ** | **digit Ⅲ** | **digit Ⅳ** | **digit Ⅴ** |
| *Hylobates* spp. | -4.3×10^-2^x+6.4894 | -5.0×10^-2^x+7.4094 | -5.6×10^-2^x+7.9265 | -4.7×10^-2^x+6.8041 |
| Adjusted R-Squared | 0.9042 | 0.9603 | 0.9549 | 0.9517 |
| *Papio hamadryas* | -1.5×10^-2^x+2.6066 | -2.4×10^-2^x+3.5172 | -3.1×10^-2^x+3.9225 | -2.0×10^-2^x+3.1482 |
| Adjusted R-Squared | 0.6644 | 0.7257 | 0.8686 | 0.8128 |
| *Ateles* sp. | -6.0×10^-2^x+7.2492 | -4.9×10^-2^x+6.5659 | -4.5×10^-2^x+6.5383 | -4.5×10^-2^x+6.6407 |
| Adjusted R-Squared | 0.9911 | 0.9828 | 0.9775 | 0.9671 |
| GEE (*p*-value) | < 0.001 | < 0.001 | < 0.001 | < 0.001 |
