# Supplementary material for: Correlation between musculoskeletal structure of the hand and primate locomotion: Morphometric and mechanical analysis in prehension using the cross- and triple-ratios
Source: PLoS One. 2020 May 4;15(5):e0232397. doi: 10.1371/journal.pone.0232397 (PMC7197777; doi:10.1371/journal.pone.0232397)
Supplement: S9 Table — (DOCX) [file pone.0232397.s022.docx]

S9 Table　 Regression equations of the corrected torque on the PIP joint angle during a suspensory hand posture

| **MCP torque** | **ID** | **Regression equation** | **Adjusted R-squared** |
| --- | --- | --- | --- |
| Hylobates | 7690 | 1.8×10^-6^x^3^-2.4×10^-4^x^2^+1.0×10^-2^x-0.10 | 0.822 |
|  | 7693 | 4.9×10^-6^x^3^-7.2×10^-4^x^2^+3.5×10^-2^x-0.50 | 0.741 |
|  | 7701 | 1.2×10^-6^x^3^-2.2×10^-4^x^2+^1.4×10^-2^x-0.18 | 0.615 |
| Papio hamadryas | 7868 | 1.4×10^-5^x^2^+2.2×10^-3^x-0.045 | 0.850 |
|  | 8300 | 9.5×10^-6^x^2^+4.9×10^-4^x+0.0036 | 0.954 |
|  | 8442 | -5.5×10^-6^x^2^+2.1×10^-3^x-0.016 | 0.912 |
|  |  |  |  |
|  |  |  |  |
| **PIP torque** | **ID** | **Regression equation** | **Adjusted R-squared** |
| Hylobates | 7690 | 7.9×10^-7^x^3^-1.1×10^-4^x^2^+5.3×10^-3^x-0.072 | 0.938 |
|  | 7693 | 1.4×10^-6^x^3^-2.1×10^-4^x^2^+1.0×10^-2^x-0.15 | 0.894 |
|  | 7701 | -2.9×10^-7^x^3^+4.0×10^-5^x^2^-1.2×10^-3^x+0.013 | 0.746 |
| Papio hamadryas | 7868 | 1.2×10^-5^x^2^-6.1×10^-5^x-0.0060 | 0.890 |
|  | 8300 | 9.6×10^-6^x^2^-5.8×10^-4^x+0.015 | 0.845 |
|  | 8442 | 1.6×10^-5^x^2^-7.0×10^-4^x+0.017 | 0.935 |
