# Supplementary material for: Correlation between musculoskeletal structure of the hand and primate locomotion: Morphometric and mechanical analysis in prehension using the cross- and triple-ratios
Source: PLoS One. 2020 May 4;15(5):e0232397. doi: 10.1371/journal.pone.0232397 (PMC7197777; doi:10.1371/journal.pone.0232397)
Supplement: S10 Table — (DOCX) [file pone.0232397.s023.docx]

S10 Table Proportion of the length of finger bones, and triple- and cross-ratios

|  | **Species** | **mc^d^** | **p^e^** | **m^f^** | **d^g^** | **m+d^h^** | **TR^i^** | **Ph CR^j^** | **MPh CR^k^** |
| --- | --- | --- | --- | --- | --- | --- | --- | --- | --- |
| A^a^ | *Hylobates lar* | 0.410 | 0.302 | 0.217 | 0.0712 | 0.289 | 1.624 | 1.167 | 1.391 |
|  | *Hylobates agilis* | 0.386 | 0.304 | 0.225 | 0.0856 | 0.310 | 1.658 | 1.189 | 1.395 |
|  | *Hylobates Syndactylus* | 0.426 | 0.306 | 0.196 | 0.0715 | 0.268 | 1.640 | 1.194 | 1.373 |
|  | *Hylobates pileatus* | 0.400 | 0.298 | 0.220 | 0.0813 | 0.302 | 1.663 | 1.184 | 1.405 |
|  | *Pongo pygmaeus* | 0.419 | 0.309 | 0.189 | 0.0829 | 0.272 | 1.688 | 1.233 | 1.369 |
|  | *Pongo abelli* (CT) | 0.416 | 0.308 | 0.191 | 0.0855 | 0.276 | 1.698 | 1.236 | 1.373 |
|  | *Ateles paniscus* | 0.352 | 0.326 | 0.218 | 0.103 | 0.322 | 1.669 | 1.238 | 1.348 |
|  | *Ateles belzebuth* | 0.399 | 0.314 | 0.201 | 0.0866 | 0.288 | 1.672 | 1.225 | 1.365 |
|  | *Ateles geoffroyi* | 0.389 | 0.307 | 0.208 | 0.0968 | 0.305 | 1.710 | 1.234 | 1.386 |
|  | *Saimiri sciureus* | 0.356 | 0.314 | 0.221 | 0.109 | 0.330 | 1.703 | 1.239 | 1.374 |
|  | *Cebus capucinus* | 0.311 | 0.313 | 0.224 | 0.152 | 0.376 | 1.797 | 1.308 | 1.373 |
|  | *Cercopithecus Diana* | 0.400 | 0.291 | 0.218 | 0.0909 | 0.309 | 1.713 | 1.202 | 1.425 |
|  | *Macaca fascicularis* | 0.410 | 0.290 | 0.196 | 0.104 | 0.300 | 1.797 | 1.262 | 1.424 |
| S^b^ | *Macaca fuscata* | 0.420 | 0.284 | 0.189 | 0.107 | 0.296 | 1.838 | 1.278 | 1.438 |
|  | *Cercopithecus neglectus* | 0.376 | 0.301 | 0.208 | 0.116 | 0.323 | 1.781 | 1.269 | 1.404 |
|  | *Pan troglodytes* | 0.421 | 0.279 | 0.202 | 0.0982 | 0.300 | 1.793 | 1.235 | 1.452 |
| T^c^ | *Gollira gollira* | 0.444 | 0.313 | 0.195 | 0.105 | 0.300 | 1.786 | 1.245 | 1.472 |
|  | *Homo sapiens* | 0.410 | 0.282 | 0.184 | 0.124 | 0.308 | 1.914 | 1.321 | 1.449 |
|  | *Papio hamadryas* | 0.480 | 0.297 | 0.193 | 0.108 | 0.301 | 1.854 | 1.317 | 1.508 |
|  | *Papio anubis* | 0.455 | 0.259 | 0.159 | 0.127 | 0.287 | 2.074 | 1.379 | 1.504 |
|  | *Theropithecus gelada* | 0.482 | 0.251 | 0.138 | 0.130 | 0.267 | 2.203 | 1.455 | 1.514 |

^a^A=arboreal, ^b^S=semiarboreal, ^c^T=terrestrial, ^d^mc=metacarpal bone, ^e^p=proximal phalanx, ^f^m=middle phalanx;

^g^d=distal phalanx, ^h^m+d=middle phalanx+ distal phalanx, ^i^TR=triple-ratio, ^j^Ph CR=Ph Cross-ratio, ^K^MPh CR=MPh Cross-ratio
