# Supplementary figures and images for: Correction: Correlation between musculoskeletal structure of the hand and primate locomotion: Morphometric and mechanical analysis in prehension using the cross- and triple-ratios
Source: PLoS One. 2020 May 21;15(5):e0233867. doi: 10.1371/journal.pone.0233867 (PMC7241756; doi:10.1371/journal.pone.0233867)

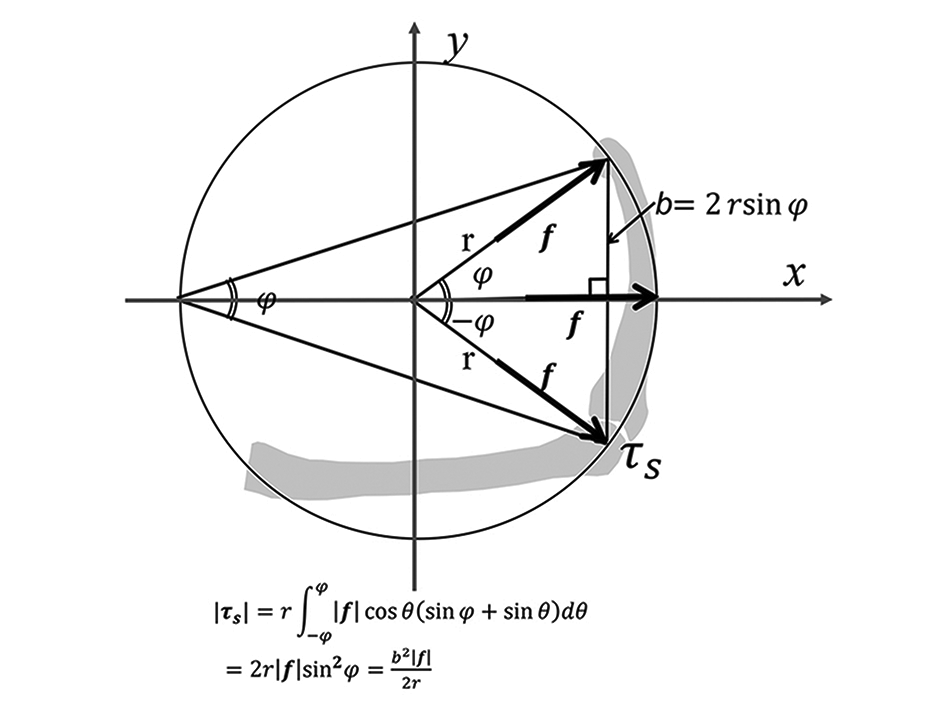

Supplement: S2 Fig — The holding torque is proportional to the square of the length of the proximal phalanx. b, length of the proximal phalanx; f, reaction force (thick arrows) from the central axis of the cylinder to the bone; r, radius of the cylinder; θ, the angle between f and x-axis; τs, joint torque. (TIF) [file pone.0233867.s001.tif]
